# Rapid Screening of Volatile Organic Compounds from* Aframomum danielli* Seeds Using Headspace Solid Phase Microextraction Coupled to Gas Chromatography Mass Spectrometry

**DOI:** 10.1155/2018/8976304

**Published:** 2018-04-19

**Authors:** Mosotho J. George, Patrick B. Njobeh, Sefater Gbashi, Gabriel O. Adegoke, Ian A. Dubery, Ntakadzeni E. Madala

**Affiliations:** ^1^Department of Chemistry and Chemical Technology, National University of Lesotho, Roma 180, Lesotho; ^2^Department of Biochemistry, University of Johannesburg, P.O. Box 524, Auckland Park 2006, South Africa; ^3^Department of Biotechnology and Food Technology, University of Johannesburg, Doornfontein Campus, P.O. Box 17011, Johannesburg 2028, South Africa; ^4^Department of Food Technology, University of Ibadan, Ibadan, Nigeria

## Abstract

Volatile organic compounds (VOCs) derived from plants have been used in the fragrance industry since time immemorial. Herein we report on the rapid screening of VOCs from seeds of ripe* Aframomum danielli* (family, Zingiberaceae) using a polydimethylsiloxane fibre headspace solid phase microextraction coupled to a gas chromatography mass spectrometry (SPME-GC/MS) instrument. Portions of 0.25, 0.35, and 0.50 g of ground sample were weighed and extraction of volatile organic compounds (VOCs) was achieved using a 100 *μ*m polydimethylsiloxane solid phase microextraction (PDMS SPME) fibre, with the equilibrium time of 40 minutes and extraction temperature of 50°C; the following compounds with their respective relative abundances were obtained as the top ten most abundant and annotated ones using NIST, Wiley, and Fragrances Libraries: eucalyptol (58%); *β*-pinene (22%); *α*-pinene (7.5%); *α*-terpineol (4%), *α*-terpinyl acetate (2%); *α*-bergamotene (1%); pinocarveol (0.39%); *α*-copaene (0.35%); caryophyllene (0.34); and *β*-bisabolene (0.31%). These compounds have been reported elsewhere in the literature and listed in the Fragrances Library, incorporated into the Saturn QP2020 GCMS Solution® software used for their analysis.

## 1. Introduction

Plants have always been part of human life where they do serve as food source not only for humans but for animals as well. There are a number of benefits that can be derived from plants other than nutritional value. Many different plants have been used in traditional medicine since time immemorial. With the increasing frequency of degenerative diseases occurrence, wild plants that have been traditionally ignored are now receiving considerable attention owing to their potential in antioxidant activity and other medical benefits thereof. As such, there is much curiosity in understanding the phytochemical composition and chemical characteristics of these herbal plants.

*Aframomum danielli, *a plant that grows widely in West Africa, is an underutilized plant species known to contain an enormous variety of interesting phytochemicals [1–3]. There are a number of reports where the* Aframomum *species demonstrated some medicinal effects such as anticancer, antiplasmodial, antiulcer, antimicrobial [4], and antifungal [5]. This plant can also act as food preservative when added to packaged foods [6]. For example, it improved the postharvest storage shelf-life of tomato [7] and has been found to stabilise the refined peanut oil more effectively than the synthetic antioxidants such as butylated hydroxytoluene and *α*-tocopherol [1]. Although different parts of this plant (flower, leaf, stem, root, and seeds) have been investigated [2, 3, 8], the seeds in particular have demonstrated very potent pharmacoactive and sensory (flavour) properties [8–10]. The seeds of* A*.* danielli* are smooth, shiny, and olive brown in colour [11] and upon crushing produce a very strong aromatic smell that resembles eucalyptus leaves, which suggests an abundance of VOCs and essential oils such as those found in* Eucalyptus* trees. Essentially, the main chemical classes of VOCs produced by plants include terpenoids, benzenoids and phenylpropanoids, alkanes, alkenes, alcohols, esters, and various derivatives of fatty acids and amino acids [12, 13].

Essentially, VOCs from plant sources are widely used in the pharmaceutical, antiseptic, flavouring, fragrance, and other cosmetic and pharmacological industries, and their analysis has been well established [14–17]. Solvent extraction and hydrodistillation are the two major conventional ways to extract VOCs from plants [17], although various other extraction methods for VOCs in different plant matrices have been reviewed in literature [17, 18]. However, there are eminent disadvantages associated with these methods, such as low recovery, destruction of sample matrix, and use of nonenvironmentally friendly organic solvents [17, 19]. Headspace solid phase microextraction (HS-SPME) is a solvent-free, nondestructive, and easy approach for collecting VOCs emitted from plants [20, 21]. This approach can be practiced even using live plants without harvesting them. Moreover, HS-SPME coupled with gas chromatography (GC) has been shown to be very efficacious, collecting considerable amounts of volatile compounds [21–24].

Herein we report the development of a screening method for VOCs using HS-SPME-GC-MS analysis from the crushed ripe seeds of* A. danielli* with the view of using some of these volatiles for possible agricultural and pharmacological applications. Different parameters, namely, temperature, amount of sample, and extraction time, were optimised followed by a qualitative and semiquantitative analysis of the most abundant VOCs obtained under the optimum conditions.

## 2. Experimental

### 2.1. Sample Collection and Preparation

Mature seeds of* A. danielli* plant used in this study were collected from the Southern region of Nigeria. The collected seeds were crushed to powder (≤0.5 mm) using a quartz mortar and pestle.

### 2.2. Sample Extraction Using HS-SPME

Different parameters amenable to headspace sampling were investigated, namely, temperature, amount of sample material used, and sampling time. SPME extraction was achieved using a 100 *μ*m polydimethylsiloxane solid phase microextraction (PDMS SPME) fibre, preconditioned for 30 minutes in a GC injection port at 200°C. Different masses (0.25, 0.35, and 0.50 g) of the ground* A. danielli* seeds were introduced into a 2 mL GC vial and the fibre was introduced 5 mm above the sample contained in the GC vial and incubated at set temperatures (20, 35, 40, and 50°C, resp.) in a water bath (Pierce, Rockford, Illinois, USA) equipped with a multivial heating unit. The incubation period was varied between 10, 20, 30, 40, and 50 minutes. After each extraction time, the fibre was retracted into the needle and introduced into the GC injection port for desorption of analytes, chromatographic separation, and subsequent detection of VOCs via mass spectrometry. The different parameters, namely, temperature, mass of sample, and the extraction time, were optimised in a univariate manner. Each extraction and GC analysis were performed in triplicate (*n* = 3).

### 2.3. Annotation of Volatiles

Tentative annotation of the analytes was achieved through the mass comparisons of the mass spectra of individual compounds and compared with the NIST 2008, Wiley 2009, and Fragrances Libraries interfaced in the GCMS Solution Software running the instrument.

### 2.4. Instrumentation

Analysis of VOCs present in* A. danielli* seeds was performed using a Shimadzu QP 2010 gas chromatograph with mass spectrometer (Kyoto, Japan) fitted with a Restek Rtx-5ms (5% phenyl-95% dimethyl-polysiloxane) capillary column with the dimensions 30 m × 0.25 mm × 0.25 *μ*m. The injection port temperature was set at 200°C and the optimised oven temperature programme began at 50°C held for 2 minutes, ramped to 170°C at a rate of 10°C/minutes, and then ramped to 250°C at a rate of 25°C/minutes and held for 2 minutes. Sample injection mode was splitless with a sampling time of 2 minutes followed by a split ratio of 1 : 10 using Helium (UHP Helium, Afrox, South Africa) as carrier gas pumped through the column at a constant flow rate of 1 mL/minute. The MS a transfer line temperature was set at 250°C, and ion source temperature was 200°C, with a scanning mode mass range of 50–500 amu.

## 3. Results and Discussion

### 3.1. Profiling and Annotation of Volatiles from the Seeds of* A. danielli*

**Figure 1:**
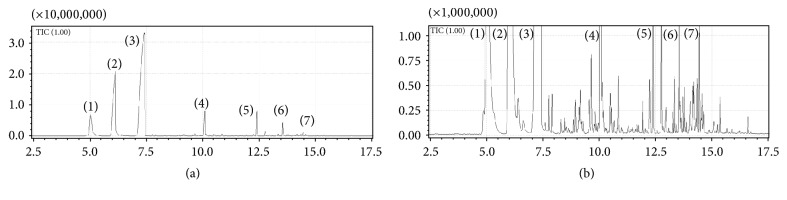
A chromatogram of the VOCs following a 40-minute extraction at 50°C: (a) depicts the chromatogram standardised against the largest peak (at about 7.5 min) expanded to cover the region between 2.5 and 17.5 minutes while (b) depicts 10 times magnified chromatogram showing several tens of peaks that are not visible in (a) due to their low relative abundances compared to the seven peaks visible in (a).

Figure 1 shows the chromatogram of the extracted volatiles from the seeds of* A. danielli* using a headspace SPME with extraction conditions of 50°C, 0.25 g of sample, and 40-minute extraction. The number annotations on the chromatogram indicates the peak indexes which correlates with their mass spectral data shown in Table 1.

It can be seen that peak (3) had the highest intensity followed by peak (2) and then peak (4). Peak (3) was annotated as eucalyptol (also known as 1,8-cineole) following confirmation on three libraries (NIST, Wiley, and Fragrances) all interfaced with the GCMS Solution software. However, a close inspection of this peak revealed that it could be composed of several compounds and not just one as shown by the small spikes at the apex of the peak as well as the different mass spectra detected at different positions of the same peak.

From Table 1 it can be seen that Wiley shows the highest match for all the compounds with NIST showing the lowest matches. However, the differences are only a few percentage points from one another. The listing of the compounds in the Flavour and Fragrances Library (FFRSC) indicates that such compounds have been used in the fragrances. Hence, this makes this plant a good candidate for use in fragrances industry.

### 3.2. The Effect of Mass of the Sample on the Production and Extraction of the VOCs

**Figure 2:**
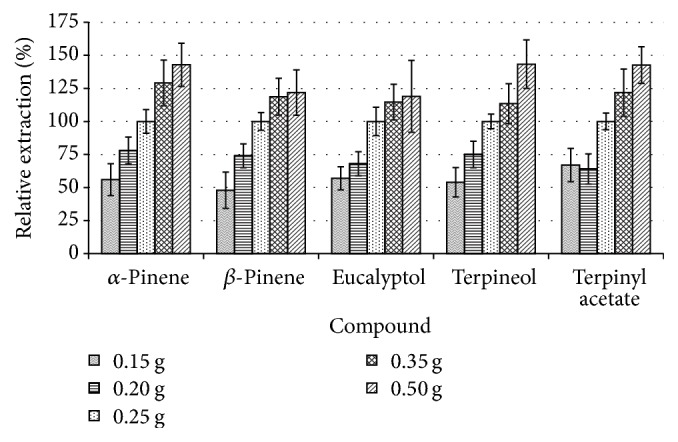
The effect of varying the amount of sample on the production of the VOCs.

Logically the increase in the amount of sample should increase the production of the VOCs in a fixed unit volume. To optimise the mass of the sample required to yield the highest amount of the VOCs, different masses of the ground seeds were used and the amount of the VOCs produced is presented in Figure 2, plotted relative to 0.25 g sample for *n* = 3 replicates.

As can be seen, the amount of the VOCs increased with the amount of the sample used. However, the increase is not linear as the data yielded the coefficient of determination, *R*^2^ ≤ 0.9126 for terpinyl acetate although visually this was the most nonlinear curve (data not shown pictorially). The extraction seems to level off beyond 0.35 g of the sample while there is also a drop in repeatability as shown by the drop in relative standard deviations from the average of 7.7% for 0.25 g sample to 18.6% for 0.5 g samples, with the abundance of the VOCs increasing to an average of 134% using 0.5 g samples relative to 0.25 g samples. The compounds that resulted in the highest VOCs production (about 140%) were *α*-pinene and the two terpenoids while *β*-pinene and eucalyptol demonstrated the lowest increase (about 120%). The levelling off could be attributed to the rate of uptake of the analytes into the fibre which in this case becomes the limiting factor. Another factor could be the saturation of the fibre; however, this is unlikely since peak areas varied considerably. If this was due to saturation then the peak areas would be almost equal at all times. This, however, cannot be argued confidently given that the analysis was restricted to a few compounds (10, although only 5 are shown on the charts for ease of visualisation), yet from Figure 1 it can be seen that there are quite a number of compounds.

Another important aspect is the increase in the deviations as the amount of the sample was increased. This could be attributed to the reduced headspace volume that resulted in the fibre touching the sample rather than being suspended in the headspace, especially with the 0.5 g samples. This negates the benefit of increasing the amount of sample since the sample particles act as variable barriers for the passage of the analytes onto the fibre surface. This is so because the particle shape and size of the ground samples were not uniform, hence having different barriers at different times.

Due to the above factors, the amount of 0.25 g was selected as the most ideal sample amount and was used for further optimisation experiments.

### 3.3. The Effect of Temperature on Extraction Efficiency of Volatiles

Temperature is one of the most universal parameters that affect the efficiency of extraction through transfer of analytes to the headspace. Recently, a solvent-assisted headspace sampling was performed using organic solvents in driving analytes into the headspace; thus considerably reducing sampling time was reported albeit in aqueous matrices [25]. To assess the effect of temperature on extraction efficiency, samples in the GC-vials in this present study were incubated at different temperatures (25–65°C) and extracted for a fixed period of 20 minutes.

**Figure 3:**
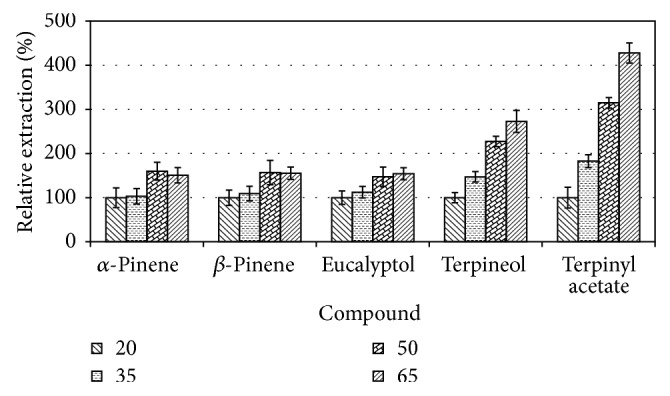
The effect of varying temperature on the extraction of volatiles from* A. danielli *seeds.

Figure 3 shows the extraction efficiency relative to room temperature (20°C) and only five compounds were shown for ease of visualisation chosen because of their abundance.

As can be seen in Figure 3, extraction efficiency of the VOCs increased 1.5-fold for the two pinene types and eucalyptol while those of terpineol and terpinyl acetate increased about 2.5 to 4.5 at 65°C compared to the 20°C (room temperature). The other interesting observation is that the latter two compounds showed the continued increase with temperature while the first three levelled off after 50°C, after which time different behaviours were noticed. This is an indication of the dynamism of SPME extraction and the increased fluidity of the polymer with increase in temperature increasing the exchange of the analytes between the fibre and the headspace volume; therefore, 50°C was chosen for further extractions. Besides, the first three compounds eluting much earlier than the last two (terpineol and terpinyl acetate) indicated that their vapour pressure is much higher and they easily saturate the headspace. The latter compounds only enrich the headspace as the temperature is increased due to their relatively lower vapour pressure.

### 3.4. The Effect of Extraction Time on Extraction of the Volatiles

**Figure 4:**
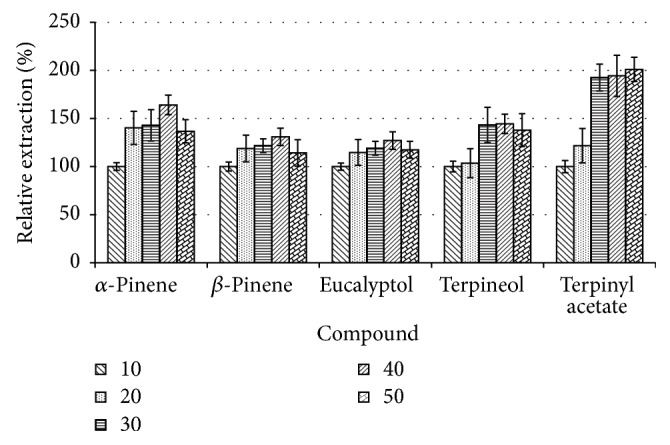
The effect of varying extraction time of volatiles of* A. danielli.*

Given the solid nature and surface area of the fibre, SPME is characterised by slower extraction kinetics taking as long as one hour than its counterpart liquid-based methods such as single-drop microextraction that typically become saturated within about 20 minutes [26]. To assess the effect of temperature and the ideal time required for the highest extraction, different 0.25 g samples of* A. danielli* were incubated at 50°C and extracted after different time periods between 10 and 50 minutes.  Figure 4 illustrates the effect of varying extraction times on the extraction of 5 most abundant volatiles recovered from the analysed spice.

As can be seen from Figure 4, the extraction of the lower volatile compounds seems to increase continuously with the increase in time (about twice that of 10-minute extraction time at 40 minutes), while for the most volatile compounds the extraction peaks and levels off at 40 minutes of extraction yielding about 1.25- to 1.75-fold that of 10-minute extraction time at 40 minutes. This trend can still be explained by the dynamism of the fibre allowing the compounds to be exchanged between the fibre and the headspace volume as alluded to in the earlier section. However, the extraction time of 40 minutes was selected as the ideal time for the extraction of these VOCs from the crushed seeds.

### 3.5. Characterisation of the VOCs by Abundance and Comparison with Reported Literature on* A. danielli* and Other Plants

Following the optimised extraction time and temperature, a semiquantitative analysis was carried out on extracts obtained at optimal temperature of 50°C for 40 minutes.  Table 2 provides a list of top 30 compounds selected on the basis of their relative abundance when extracted under optimised conditions as well as comparison with those reported in literature from the same plant (seeds, etc.).

Clearly, it can be seen that the volatiles are dominated by compound depicted as peak (3) assigned as eucalyptol accounting for about 60% of the total peak areas for 30 peaks integrated automatically using autointegration software. This is followed by *β*-pinene oxide at 22%, *α*-pinene at 7.5%, and terpineol at 4% as well as terpinyl acetate and bergamotene that accounted for about 2 and 1%, respectively, with all the 26 remaining compounds, each with less than a percentage point collectively taking the remaining 6.5%. However, it must be noted that the percentage indicated for each compound is relatively skewed due to the abundance of the first three compounds; most of these compounds are still significantly abundant as can be depicted in Figure 1.

## 4. Conclusions

The presented work demonstrated the effectiveness of the proposed method for screening the VOCs from the* Aframomum danielli* which in essence demonstrated the applicability to other plant seeds with rich content of volatile oils. In the current study, the amount of seeds material, time of extraction, and temperature of extraction were found to affect the outcomes of the extracted metabolites, both qualitatively and quantitatively. Thus, 0.25 g of finely ground ripe seeds, extraction time of 40 minutes, and a temperature of 50°C were found to be optimal for the extraction of the reported metabolites. Our results suggest that* A. danielli* seeds contain eucalyptol as a dominant VOC (about 60% of all the VOCs detected) with only six (6) compounds accounting for almost 95% of the total VOCs produced. Overall, A*. danielli* can be regarded as a potent source of eucalyptol oil; hence this plant can be used as a potent source for commercial production of this eucalyptol oil.

## Figures and Tables

**Table 1:** Some chromatographic data and tentative annotation of the top ten most abundant VOCs obtained from the headspace extraction of the ground *A. danielli* seeds.

Peak	Retention time (min)	Compound name	RMM	Ref Ion	Qual Ion	Different library matches (%)		
						NIST	FFSC	Wiley
(1)	5.087	*α* -Pinene	136	93	77	96	96	97
(2)	6.086	*β*-Pinene	136	93	69	96	96	97
(3)	7.259	Eucalyptol	154	81	154	91	89	91
(4)	10.082	*α*-Terpineol	154	59	136	94	94	96
(5)	12.434	*α*-Terpinyl acetate	196	121	93	93	95	96
(6)	13.515	Bergamotene	204	119	93	95	95	96
(7)	14.445	*β*-Bisabolene	204	69	93	90	95	96

*Key*. RMM: relative molecular mass; Ref Ion: reference ion; Qual Ion: qualifier ion.

**Table 2:** Top 30 most abundant VOCs extracted from *A. danielli* seeds annotated using the three libraries listed in Table 1.

Retention time (min)	Percentage abundance	Annotations^*∗*^	Literature reported (*Aframomum* spp.)	Literature reported (other plant species)
4.02	0.15	*α*-Thujene	[27, 28]	
4.134	7.58	*α*-Pinene	[28, 29]	
4.898	22.11	*β*-Pinene oxide		[30]
5.886	58.10	Eucalyptol	[29]	
6.173	0.08	*γ*-Terpinene	[31]	
6.294	0.09	Sabinene hydrate 〈E〉^*δ*^	[28, 32]	
6.768	0.08	Sabinene hydrate 〈Z〉	[28]	
7.187	0.12	*α*-Campholenal	[31]	
7.388	0.39	Pinocarveol 〈Z〉		[33]
7.743	0.11	Pinocarvone	[28, 34]	
7.823	0.28	*δ*-Terpineol	[31]	
7.976	0.11	Terpinen-4-ol	[34]	
8.235	4.08	*α*-Terpineol	[28, 34]	
8.594	0.21	Carveol 〈Z〉	[28]	
8.941	0.18	Carvone	[28]	
10.271	0.21	Carvyl acetate 〈Z〉		[35]
10.419	1.83	*α*-Terpinyl acetate	[28]	
10.788	0.35	*α*-Copaene	[28, 29]	
10.988	0.11	*β*-Elemene	[28, 36]	
11.359	0.12	Caryophyllene 〈(E)〉		[37, 38]
11.553	1.17	*α*-Bergamotene 〈Z〉	[34]	
11.73	0.09	Valerena-4,7(11)-diene		[39]
11.789	0.12	*β*-Bergamotene 〈E〉	[28, 32]	
12.05	0.15	Aciphyllene^#^		[40–42]
12.168	0.34	Caryophyllene (Z)		[38]
12.356	0.21	Isodaucene		[43]
12.446	0.31	*β*-Bisabolene	[29, 34]	
12.571	0.19	*β*-Selinene	[29, 34]	
13.365	0.13	Caryophyllene oxide	[34]	
14.493	0.07	*α*-Bergamotol 〈(Z), E〉		[44]

^*∗*^Annotation was made with the match of ≥85% from the NIST Library, with the lowest match of 85% obtained for the last three entries (sabinene hydrate 〈E〉, *γ*-terpinene, and *α*-bergamotol) with percentage abundances between 0.07 and 0.08%. ^*δ*^The E isomer has been reported elsewhere to elute earlier than the “Z” isomer [28, 32]. ^#^Also reported in bacteria.
